# The Psychology of Atopic Dermatitis

**DOI:** 10.3390/jcm13061602

**Published:** 2024-03-11

**Authors:** Ashling Courtney, John C. Su

**Affiliations:** 1 Department of Dermatology, Eastern Health, Monash University, Melbourne, VIC 3128, Australia; 2 Murdoch Children’s Research Institute, Melbourne, VIC 3052, Australia

**Keywords:** atopic dermatitis, eczema, itch, mental health, depression, alexithymia, obsessive–compulsive disorder, suicide, inflammation, dementia, cognitive impairment

## Abstract

Atopic dermatitis (AD) is a common chronic inflammatory skin condition characterised by pruritus and recurrent eczematous patches and plaques. It impacts sleep and its visibility can lead to stigmatisation, low self-esteem, social withdrawal, reduced quality of life (QOL), and psychological burden. This study explores the relationship between AD and mental health, including possible causation pathways. A literature review was conducted in PubMed without using limiters. AD carries higher odds of suicidality and an increased risk of depression, anxiety, alexithymia, and obsessive–compulsive disorder (OCD) across all severities. While some studies report an association of AD with attention deficit hyperactivity disorder (ADHD), and possibly autism spectrum disorder (ASD), others do not. There is increasing evidence that AD contributes to chronic low-grade inflammation and cognitive impairment (CI). Causative factors for mental health complications of AD likely include both psychosocial and biological variables. AD is associated with higher levels of cutaneous and circulating proinflammatory cytokines; these can breach the blood–brain barrier and trigger central nervous system events, including oxidative stress, neurotransmitter breakdown, altered serotonin metabolism, and reduced neurogenesis in several brain regions. Excessive inflammation in AD may thus contribute to CI, depression, and suicidality. AD providers should be vigilant about mental health.

## 1. Introduction

AD is a chronic inflammatory skin condition characterised by relentless pruritus and recurrent eczematous plaques [1]. It can severely impact sleep, schooling, work, and family relationships; visible disfigurement from redness, flaking, and bleeding excoriations can result in social stigmatisation, withdrawal, and reduced QOL [2,3]. The financial strain, both personally and socially, is also high [4]. 

AD is the leading cause of skin disease worldwide, affecting approximately 20% of children and 10% of adults [5]. It is caused by complex interactions between genetic, environmental, and socioeconomic factors [1]. Important determinants include skin barrier dysfunction, neuroinflammation, immune dysregulation/activation especially of the Th2 axis, microbiome abnormalities, noxious environmental stimuli, and genetic variations (particularly of filaggrin and immune activation-associated genes) [6]. Immune dysregulation and activation has also been associated with depression in several patient populations [7].

Personalised AD treatment includes the identification and addressing of particular psychosomatic, allergic, and environmental issues [8]. Psychosocial associations of AD include a depressed mood, shame and disgust, insomnia, social anxiety, stigmatisation, compulsive scratching, and neuropsychiatric conditions including depression, anxiety, OCD, and suicidal ideation [9,10,11]. Both the chronicity and visibility of AD render children at risk for strained family relationships, poor self-image, bullying, and psychiatric comorbidities [12,13,14].

However, it remains unclear whether neuropsychiatric comorbidities in AD result from persistent and intense itching, poor sleep, and stigma, or if they are direct consequences of proinflammatory cytokines generated by AD inflammation and accelerated neuroinflammation [7,15]. The contributory role of sleep disturbance and itch in the development of AD psychosocial complications has been studied, but the roles of psychological, neurological, and molecular factors are, to date, largely unquantified [16,17].

There are reports of an increased risk of ASD and ADHD in infants with AD, suggesting that an immune dysregulation may influence neurodevelopment [18,19,20]. Additionally, evidence suggest that a higher expression of pro-inflammatory cytokines in ageing skin may be associated with ageing-related CI [21]. This review explores the associations between AD, mental health, cognitive function, and possible causation pathways between chronic cutaneous inflammation, itch, and psychological wellbeing. 

A search was conducted in Pubmed using the keywords ‘atopic dermatitis’, ‘eczema’, “mental health”, “neuroinflammation”, “depression”, “anxiety”, “obsessive–compulsive disorder”, “alexithymia”, “neurodevelopmental disorder”, “cognitive impairment”, and “causation pathways”. No limiters were applied. There was a focus on the literature published within the last five years. A summary of important findings from selected publications included in this review are available in the Supplementary Materials, Table S1.

## 2. Mental Health Conditions Associated with AD

AD has been associated with depression, anxiety, ADHD, CI, and suicidality. Quantifying risk has been difficult due to inconsistencies between studies in diagnostic definitions of mental health disorders and study failures to consider AD’s severity [22]. In a scoping 30-year-period literature review, Zeiser et al. found that the effect of depression as a consequence of AD severity is stronger than the effect of depression on AD as an exacerbating factor [23]. Adults with AD are more likely to develop new depression and anxiety, and a dose-response relationship with AD severity has been observed for depression [9]. A systematic review and meta-analysis on the association of AD with depression, anxiety, and suicidal ideation, including studies from Europe, Asia, and North America, found that the relationships are independent of nationality [22]. It also noted that data from Africa and South America are lacking.

The cycle of itch, poor sleep, poor self-image, psychologic stress, social isolation, depression, and anxiety can self-perpetuate. In severe AD, this may result in suicidal ideation, attempts, and in some cases completed suicide [22]. In a meta-analysis on the association between AD and suicidality, patients with AD were 44% more likely to exhibit suicidal ideation and 36% more likely to attempt suicide compared to patients without AD [11]. A large UK-based cohort study comparing 625,083 adults with AD to 2,678,888 adults without AD showed that AD was associated with a higher risk of anxiety [hazards ratio (HR) 1.14 (95% confidence interval (CI) 1.13–1.15)], depression [HR 1.14 (95% CI 1.13–1.15)] and OCD [HR 1.48 (95% CI 1.38–1.58)] across all severities [15]. Among children/adolescents, females have shown a higher risk for suicidal intention, planning, and attempts [24]. It is important for dermatologists to be aware of this risk in patients with AD, screen for depression and suicidality, and make referrals when necessary.

### 2.1. Depression

The prevalence of depression for individuals with AD is estimated at 20%, corresponding to a two-fold risk compared to the general population and a four-fold risk of suicidal ideation compared to those without depression. [22,25,26]. More parents (29.3% vs. 20.3%) of affected children also report depression. Depressive symptoms in patients with inflammatory skin diseases are often attributed to the psychosocial impact of living with a chronic visible illness, and for AD, are specifically attributed to chronic itch, poor sleep and self-image [12]. However, symptoms may also be driven by underlying cutaneous inflammation [7]. Inflammatory markers are often raised in major depressive disorder [7] and microglial inflammation potentially induces major depressive episodes [27]. 

AD is characterised by skin inflammation and associated with higher cutaneous and serum levels of proinflammatory cytokines, such as interleukin (IL)-4, IL-13, IL-22, thymic stromal lymphopoietin, IL-33, and variably, IL-17 [6]. It has been suggested that IL-13, which binds dopaminergic neurons and stimulates astrocyte production of brain-derived neurotrophic factor, with oxidative stress, which may contribute to neuronal damage in the ventral tegmental and substantia nigra (rather than serve a neuroprotective function) and may predispose an individual to depression and suicidality [28,29]. Improved mental health outcomes are associated with newer targeted therapies for chronic inflammation (e.g., IL-4, IL-13, and Janus Kinase inhibition), but it is unclear how much of this results through direct or indirect means [30,31]. 

Several mechanisms may allow inflammation to contribute to depression and depressive symptoms [7]. Elevated serum levels of other circulating cytokines, such as interleukin (IL)-6 and tumour necrosis factor (TNF-α) (depending on the depression subtype), have also been detected during major depression, showing a dose-response relationship with the severity of depression. IL-6 and TNF-α may also compromise the blood–brain barrier in depression [32]. Increased levels of TNF-α accelerate the breakdown of neurotransmitters, such as serotonin, which are deficient in depression; this may potentially contribute to the association with suicidality in patients with AD [6]. In addition, central inflammation has been associated with oxidative stress and reduced neurogenesis, which are implicated in depression. A bidirectional relationship between physiological inflammatory responses and psychological stress may predispose susceptible individuals both to depression and further inflammation [7]. In addition to these mechanisms, itch and insomnia also mediate the effects of AD on mental health.

Itch–psyche interactions and psychosocial factors in AD have been described in the *moi-peau* concept [33,34]. This concept suggests that the skin holds significant psychological meaning, reflecting various needs of the psyche. First, the skin functions as a protective “container” for positive stimuli in infancy. Second, it acts as an “interface”, guarding against external threats. Third, skin serves as a unique “place” for communication and a “surface” for interpersonal connections [33,34]. Therefore, chronic skin diseases acquired in early childhood can disrupt both physical and psychological wellbeing. However, the roles of neurological pathways, biological determinants of itch perception, and other factors affecting the impact of itch on affect, attention, and expectancy have been less studied [35]. In AD patients, the posterior cingulate cortex and praecuneus have shown a higher reactivity to itch stimuli [36], and the frontostriatal circuit may have a particular role in the processing of contagious itch [37]. Regions including the amygdala, hippocampus, anterior cingulate/insular cortex, mid-cingulate cortex, and prefrontal cortex also have important roles in itch processing; these are also related to emotional responses, including fear, anxiety, memory, and stress regulation [38]. The relationship between itch processing and mood, with respect to these anatomical pathways, require further study.

Of sixty children aged 1–4 years old with moderate-to-severe AD, 86% had significantly reduced sleep. Fifty percent had five or more sleep-disturbed nights each week [39] and parents also experienced loss of sleep [40]. Sleep disturbance is seen in 33–90% of adults with AD [41]. Anxiety, depressive symptoms, and CI (of attention, concentration, memory, and coordination) are well described effects of insomnia. Changes in the left orbitofrontal and right middle temporal cortex, praecuneus, posterior cingulate cortex, and thalamus have been associated with insomnia [42]. Both the exact role of insomnia in the mediating effects of AD on mental health and the potential benefits of sleep therapy require clarification.

In a systematic review assessing the use of oral antidepressants in chronic pruritus (not exclusively of AD), most articles demonstrated a significantly reduced pruritus during treatment [43]. Oral antidepressants evaluated in these studies included SSRIs (e.g., fluoxetine, fluvoxamine, paroxetine), sertraline, tricyclic antidepressants (e.g., amitriptyline, nortriptyline, doxepin), and the atypical tetracyclic antidepressant, mirtazapine [43]. Typically, due to their favourable safety profile, SSRIs are considered the primary pharmacological choice for treating depression, anxiety, and OCD in the elderly, adult, and paediatric populations [44,45,46,47]. Alternative preferred options in adults include tricyclic antidepressants, mirtazapine, bupropion, and venlafaxine [44]. Depression in the elderly can be more challenging to diagnose and treat, frequently requiring specialist input and support from caregivers. Pharmacological management is based on past treatment response and adverse effects, comorbidities, and patient/family preferences. The SSRIs, escitalopram and sertraline, are considered to have fewer drug interactions [47]. Non-pharmacological management includes psychoeducation and psychotherapeutic interventions such as cognitive behavioural therapy (CBT) and interpersonal therapy (IPT). Combination therapy using pharmacological and non-pharmacological interventions may be more effective than monotherapies for depression [44,47,48]. These treatments may therefore have a role to address anxiety, depression, or OCD in AD to benefit mood, itch, the itch–scratch cycle, sleep, and mental wellbeing.

### 2.2. Obsessive–Compulsive Disorder

OCD is a chronic mental health disorder characterised by persistent, unwanted thoughts and repetitive behaviours or mental acts. Recently, increased OCD among those with AD was shown in both a large, nested case-control study of American adults (odds ratio 2.00 (CI 1.68–2.39)) and a longitudinal study of British children (adjusted hazard ratio 1.26 (CI 1.16–1.37)) [49]. Persistent itching in chronic AD is in itself not OCD but may trigger repetitive or compulsive behaviours. Conversely, compulsive hand washing, often observed in OCD, may contribute to the worsening of AD [49]. Inflammatory processes may play a primary or secondary role in OCD [50]. Consequently, peripheral and systemic inflammation in AD could potentially escalate neuroinflammation, predisposing individuals to OCD and other neuropsychiatric disorders [10].

Effective treatments for OCD in adult and paediatric patients include behavioural therapy (exposure and response/ritual prevention and CBT) and serotonin-reuptake inhibitor (SRI) pharmacotherapy [45,46]. The latter includes clomipramine (a nonselective serotonin–norepinephrine reuptake inhibitor with preferential serotonergic action), and selective SRIs (SSRIs), including sertraline, fluvoxamine, fluoxetine, paroxetine, citalopram, and escitalopram [45]. A combination of in-person CBT and SRIs may increase efficacy, but paediatric studies remain limited and treatment personalisation is important [45,46].

### 2.3. Alexithymia

Alexithymia describes a psycho-affective construct characterised by a difficulty in identifying, expressing, and understanding one’s own emotions [51]. Individuals with alexithymia may struggle to recognise and articulate their feelings, making it challenging for them to communicate and connect with others on an emotional level. This condition can affect interpersonal relationships and may be associated with various mental health issues. In the general population, alexithymia’s prevalence is approximately 10% [51]. It may act as a triggering factor for many medical and psychiatric disorders and is associated with worse outcomes and heightened psychosocial comorbidities [51]. 

The prevalence of alexithymia in AD, up to 67%, is higher than in controls [51]. Borderline alexithymia is also more prevalent in AD [52]. Using the 20-item Toronto Alexithymia Scale and the Beck Depression Inventory (BDI) questionnaire, Talmonti et al. (2021) found both TAS-20 and BDI scores were elevated in AD patients [2]. Chiricozzi et al. (2020) showed that alexithymia was more common among patients with severe (43.6%) compared with mild AD (15.6%) and this correlated with itch intensity and sleep disturbances [52].

It is unclear if and how alexithymia might contribute to AD and AD severity; research is limited [53]. A complex neural–immuno–cutaneous–endocrine network may act as an independent local stress response system [53,54,55]. Alexithymia and subjectively perceived helplessness appear to positively correlate with post-traumatic stress, but the nature and direction of causality is unclear, as is also the case for alexithymia and AD [56].

Higher baseline sympathetic activity with higher heart rate and electrodermal activity and lower oxygen consumption has been found in alexithymic persons [57]. Alexithymia may also be linked to altered immune responses. In susceptible individuals, neuroimmune substances, such as neuropeptides, triggered by stress, can lead to increased cutaneous inflammation, an impaired epidermal barrier, and increased itch [54,55,58]. Guilbaud et al. hypothesised that alexithymia may physiologically resemble a chronic stress condition with up-regulated T helper 2 (T_H_2) and impaired T helper 1 (T_H_1) responses, while individuals remain emotionally unaware of their true distress [57]. Supporting this theory, Corcos et al. identified a positive association between the serum levels of interleukin-4, a T_H_2 cytokine, and elevated alexithymic scores, proposing that a cytokine imbalance might contribute to both psychological and somatic consequences [59].

Conversely, the cause of alexithymia is unclear, although it has been variably related to altered structure, functioning, or connectivity of regions associated with emotional processing such as the insula, anterior cingulate cortex, amygdala, and prefrontal and orbitofrontal cortex [60]. Cutaneous mechanoreceptor dysfunction has been recently associated with alexithymia, but the effect of AD-related itch on alexithymic neural pathways remains unexplored [61]. 

Additional potential interactions between AD and alexithymia are those mediated by other psychosocial problems affected by alexithymia, which may in turn affect AD treatment outcomes [51]. These include social anxiety, social avoidance., depression, and interpersonal difficulty, all of which may contribute to AD undertreatment and severity [62,63,64]. Together with low self-esteem, stigmatisation, and low expectations for interpersonal and intrapersonal change, alexithymia has also been found to correlate with low motivation for psychotherapy [65]. Stigmatisation in AD involves both stigmatisation by others and self-stigmatisation. Stigma, esteem, efficacy, depression, and activation are inter-related and any therapy should address all of these; otherwise, low motivation and hopelessness would deter any possibility of therapeutic progress [66,67,68].

Studies examining the treatment of alexithymia in dermatological patients are few, but successful outcomes following group therapy, journaling, emotion labelling, structured exercises, and psychoeducational and skills training have been described for neurological, oncological, psychiatric, and surgical patients irrespective of the severity of alexithymia or of patient suffering [51]. Sustained reductions in TAS-20 scores and improvements in medical conditions have thus been observed [51]. Studies of AD and psoriasis patients with alexithymia have shown that alexithymia may be reversible with effective treatment of the skin [51,69]. Additionally, the reversal of alexithymia is correlated with enhancements in measures of disease severity, psychological comorbidities, work productivity, and overall QOL [69]. 

### 2.4. The Psychological Effect of Atopic Dermatitis in Childhood

AD is one of the commonest chronic diseases of childhood and adolescence [8]. In infants and young children, chronic, severe AD is associated with a risk of failure to thrive; however, not much is known about the neurodevelopmental and neuropsychiatric effects of AD on children [8]. Most children with AD have difficulty falling asleep and sleeping through the night, leading to daytime sleepiness, which in turn can affect concentration and performance at school [8]. Additionally, children with AD often face teasing and bullying from their peers at school due to negative misconceptions or misinformation regarding AD, and this stigmatisation can have harmful effects on their education and psychosocial development [12]. A large UK population-based cohort study compared children with AD (93.2% mild, 5.5% moderate, 1.3% severe) with children without AD and found no statistically significant relationship between AD and incident anxiety, ADHD, ASD, bipolar disorder, suicidal ideation, and attempt or completed suicide [49]. Children with AD were less likely to develop depression or schizophrenia but more likely to develop OCD. However, the study acknowledged that there was substantial variation in AD severity and age in both the direction and magnitude of effect for many of the conditions examined [49]. In a Korean study, adolescent patients with AD were at greater risk of suicidal ideation and suicide attempts [70]. Another more recent Korean study of 788,411 adolescents found that female patients with AD had an increased risk of suicidal ideation and suicide attempts compared with healthy controls [71]. Among the 22% of adolescent patients with AD, 34.7% reported depression, 19% reported suicide ideation, and 4.5% reported suicide attempts. Perceived stress and unhappiness related to their AD were the most influential factors given for depression and suicidal ideation [71]. The pathogenesis of depression and suicidality in patients with AD may differ between adults and children. Furthermore, maturation of the paediatric brain may be influenced by the systemic type 2 immune response in AD [72]. During early infancy, the blood–brain barrier is more permeable and systemic type 2 inflammation may impact fatty acid elongation and composition in the brain, but this has not been proven [72].

AD has reportedly been associated with ADHD and ASD [18,19,20]. However, evidence in the literature is conflicting [49]. A recent systematic review and meta-analysis of the association of AD with ADHD and ASD suggested that patients with AD have 1.28-fold increased odds of ADHD and 1.87-fold increased odds of ASD when compared to those without AD [20]. The odds of ADHD in patients with severe AD (pooled OD 3.46 (95% CI 2.25–5.30)) were statistically significantly higher than those in patients with mild-to-moderate AD (pooled OD 1.31 (95% CI 1.09–1.590)) [20]. In addition, the odds of ADHD were significantly greater in studies enrolling school-age children and adolescents, 6 to 18 years (pooled OD 1.38 (95% CI 1.28–1.48)), when compared with studies evaluating preschoolers, age <6 years (pooled OD 1.38 (95% CI 1.28–1.48)) [20]. The pooled prevalence of ADHD and ASD in patients with AD were 6.6% and 1.6%, respectively, whilst the prevalence of ADHD and ASD in the general population were approximately 2.8% and 1%, respectively [20,73,74]. U.S. cohorts suggest an increased risk of ADHD corresponding with AD severity, stating adjusted odds ratios of 1.56 (CI 1.22–1.99) for mild–moderate and 16.83 (CI 7.02–40.33) for severe AD with three or less nights of satisfactory sleep in a week [75,76]. However, the association with ASD remains unclear. Two Taiwanese cohort studies identified a greater risk of ASD and ADHD in children with AD compared with those without AD [18,19]. Although, the estimates of ASD ranged widely from 10% to 900% higher. Both studies concluded that children diagnosed with AD before age 3 are at an increased risk of developing ADHD and ASD later in childhood. Additionally in a few paediatric cohort studies, children with AD had a 16–300% increased risk of ADHD compared to the general population [18,19,49]. Other studies have found no overall association between AD and ASD or ADHD [49,77]. 

AD, ADHD, and ASD share seemingly similar pathomechanisms involving inflammation, genetics, and the microbiome [20]. Consequently, several hypotheses have been described to illustrate the positive associations between AD and neurodevelopmental disorders [20].

First, in AD, mast-cell-driven vasoactive mediators may heighten the permeability of the blood–brain barrier [78]. This phenomenon allows pro-inflammatory cytokines to traverse the blood–brain barrier causing possible focal brain inflammation, which might influence synaptic plasticity, subsequently affecting the development of behavioural disorders such as ASD and ADHD [79]. Neuroinflammation in AD may also modify neurotransmitter metabolism [80]. This proposed mechanism aligns with some previously discussed findings, which reveal a higher likelihood of ADHD for severe AD compared with mild-to-moderate AD [19,20]. Consistent with this, Liao et al. reported an increasing ADHD risk corresponding with increasing AD severity (*p* < 0.001) [19].

Second, the dysregulation of DNA methylation may play a pivotal role in both AD and ADHD. Studies have identified hypomethylation in patients with both AD and ADHD, indicating that genetic factors may be associated with disease risk through epigenetic pathways [81,82].

Additionally, previous research has reported phenotypic overlap and similar mRNA expression patterns between AD and ASD [83].

Third, given the discovery of an aberrant gut microbiome in AD patients, dysbiosis may contribute to brain dysfunction by disrupting the gut–brain axis [84,85]. More studies are needed.

AD, however, is associated with increased risks of emotional and behavioural problems and may also affect cognitive function [22,86,87]. Studies examining the relationship of AD with school performance and cognitive function among children and adolescents are however limited and inconsistent [86,87]. A recent Danish cross-sectional study examining hospital-managed AD in adolescents and young adults found that severe AD was associated with poorer school performance in early adolescence and with a lower IQ in young men [88]. However, the results of a longitudinal study in the UK evaluating the relationship of AD activity and severity with validated measures of general cognition did not find any clinically meaningful associations [86]. Moreover, AD-related symptoms, which are thought to impact adolescents’ mental health through pathways including disturbed sleep, impaired self-esteem, social isolation, and concentration difficulties, may have negative repercussions on school performance and cognitive function. Stress from poor school grades could therefore worsen AD symptoms, creating a vicious cycle [22,87]. Future longitudinal studies are needed to explore the relationship between AD and academic performance [87].

CBT is a recommended initial intervention for anxiety disorders in children and adolescents [48]. For depressive disorders, treatment guidelines suggest either CBT or IPT as the primary treatment methods [48]. SSRIs are usually the most effective pharmacological treatment for anxiety and depressive disorders in paediatric patients. Combined therapies, which involve both psychotherapy and an SSRI, have demonstrated superior therapeutic effects compared to either treatment alone [48]. In children, the combination of CBT and sertraline has been advocated for anxiety, while pairing CBT or IPT with fluoxetine has been recommended for depression [48]. These combination therapies are particularly valuable for patients exhibiting an insufficient response to treatment with just an SSRI or psychotherapy [48]. 

ADHD pharmacological treatment includes stimulant medications like methylphenidate and d-amphetamine, and nonstimulants such as atomoxetine [89]. These drugs are believed to exert cognitive-enhancing effects through the modulation of noradrenaline and dopamine. However, the specific mechanisms responsible for these effects remain unclear. In addition, changes in diet, exercise, sleep, screen time limits, and nature exposure may benefit both symptom relief and long-term control [48].

### 2.5. ‘Inflammaging’ and Cognitive Impairment in the Elderly

There is increasing evidence that AD severity contributes to cognitive dysfunction or impairment in adults [90,91,92]. CI is a symptom of neurodegenerative disorders and can be a precursor for dementia and Alzheimer’s disease [92]. It can manifest as difficulties with memory, attention, language, perception, or executive functions. The exact cause of CI is unclear; however, epidemiological studies have shown that the incidence of CI is higher in subjects with certain inflammatory skin disorders, including chronic eczema [92]. 

Individuals aged over 65 years may display chronic, low-grade, generalised inflammation, commonly referred to as ‘inflammaging’, even without a specific inflammatory disorder [21,92,93]. The cause of ‘inflammaging’ likely involves immune system dysregulation and various other age-related physiological changes [94]. This has been linked to the development of many aging-associated systemic disorders, including type 2 diabetes, obesity, cardiovascular diseases, cancers, and certain neurodegenerative disorders [21]. Age is also associated with epidermal dysfunction. One study of 255 participants aged ≥ 65 years found that stratum corneum hydration levels on both the forearm and the shin correlated negatively with serum cytokine levels [93]. The reduced stratum corneum hydration with skin barrier disruption can induce inflammation and contribute to ‘inflammaging’ [92,94]. In addition, the skin of AD patients shows a predominance of T-helper-2-derived cytokines which may also contribute to stress-induced inflammation in AD [7]. As previously discussed, pro-inflammatory cytokines, such as TNF-α, IL-6, and dendritic cells, which are important drivers in the pathogenesis of AD, can breach the blood–brain barrier and trigger a cascade of events in the central nervous system, including oxidative stress, breakdown of neurotransmitters, and reduced neurogenesis in several brain regions [7]. Hence, excessive inflammation in the skin of elderly patients, such as in AD, may contribute to cognitive dysfunction and decline [7,92,94].

Assessing cognitive function could serve as an important outcome measure for evaluating treatment response in AD [90]. A single-centre study of 188 adult patients with AD showed that over 58% had at least one or more symptoms of cognitive dysfunction in the preceding 4 weeks [90]. Cognitive function scores were inversely associated with all patient and physician eczema measures with stepwise decreases in cognitive function with worsening AD severity [90]. A longitudinal Taiwanese study of 1059 patients with AD and 1:10 matched controls (*n* = 10,590) found that patients with AD were more likely to develop any dementia, particularly Alzheimer’s disease, than those in the control group. Moderate–severe AD was associated with a high subsequent dementia risk and AD may be an independent risk factor for new-onset dementia [91]. Another longitudinal study found that a history of atopy was associated with a 16% increased risk of Alzheimer’s disease (HR = 1.16; CI 0.98–1.37) or any dementia (1.16; 1.01–1.33) in a case-control analyses of twins adjusting for age, sex, educational attainment, smoking history, and myocardial infarctions [95]. Furthermore, the twin with a history of eczema had a 96% increased risk of developing Alzheimer’s disease compared to the healthy co-twin (HR = 1.96; 1.06–3.62) [95]. In monozygous twins, the HR for eczema increased to over four for both Alzheimer’s disease (4.47; 0.93–21.46) and any dementia (4.18; 1.35–12.97) [95]. Although current medications cannot cure Alzheimer’s disease, U.S. Food and Drug Administration-approved treatments that may help lessen symptoms, such as memory loss and confusion, include donepezil, rivastigmine, galantamine (acetylcholinesterase inhibitors), and memantine (NMDA inhibitor) [96]. However, most drug half-lives are short, and their adverse effects can be significant [96]. Strategies such as biologic agents that improve epidermal function can lower cytokine levels in both the skin and circulation and may help to alleviate depressive symptoms and aging-associated disorders such as mild CI [7,92]. Thus, it seems likely that cutaneous inflammation in AD contributes to ‘inflammaging’ and CI in the elderly. 

## 3. Conclusions

AD is a debilitating skin condition with a heavy psychological burden. Increasing evidence shows a positive association between AD and several neuropsychiatric disorders. Studies have shown that the effect of depression as a consequence of AD severity is stronger than the effect as an exacerbating factor [23]. Despite advances in the treatment of AD, the nature of mental health challenges faced by AD patients, their pathogenesis, and ways to optimise patient engagement and life quality are still inadequately understood. Children with AD face multiple challenges including poor sleep, self-image, stigmatisation, and strained family relationships, which can adversely affect their normal development. Prospective studies are required to clarify neuropsychological variables and complications for children with AD. Oral antidepressants may be considered in patients with AD-related chronic pruritus that is unresponsive to topical or systemic treatment, but further studies including randomised-controlled trials are required [43]. The predisposition of severe AD to ‘inflammaging’, impaired cognition, and new-onset dementia and potential benefits of early barrier correction in the elderly also needs examination [91]. The timely screening and referral of AD patients of all ages at risk of mental co-morbidities are most important. 

## Data Availability

The data are contained within the article.

## Supplementary Materials

The following supporting information can be downloaded at: https://www.mdpi.com/article/10.3390/jcm13061602/s1, Table S1: Summary of important findings from selected publications included in this review.
