# Supplementary material for: The Psychology of Atopic Dermatitis
Source: J Clin Med. 2024 Mar 11;13(6):1602. doi: 10.3390/jcm13061602 (PMC10970750; doi:10.3390/jcm13061602)
Supplement: Supplementary file 1 [file jcm-13-01602-s001.zip › jcm-2889662-supplementary.pdf]

Supplemental Table S1. Summary of important findings from selected publications included in this review.

Reference [no.]<br><br>Title	Objective	Study design/Type of article	Sample size, characteristics	Mental health condition assessed	Findings
Talamonti et al [2]<br><br>Quality of Life and Psychological Impact in Patients with Atopic Dermatitis	To determine the degree of psychological stress in patients with AD compared to healthy participants.	Cross-sectional	Total of 352 adult patients<br><br>174 with AD<br><br>178 healthy participants	Alexithymia<br><br>Depression	• High TAS-20 and BDI scores among patients with AD<br>• The prevalence of alexithymic personality features was 56.3% in patients with AD versus 21.3% in healthy controls [$p < 0.001$]. Based on BDI scoring [BDI-21 > 13], depression was suspected in a significantly higher number of patients with AD than in the control group [56.9% [99/174] vs. 15.7% [28/178]; $p < 0.0001$]
Frazanfar et al [7]<br><br>Inflammation: A Contributor to Depressive Comorbidity in Inflammatory Skin Disease.	The published literature from 2012-2017 was reviewed for evidence of a relationship between depression and inflammatory processes in the context of skin pathology	Review	NA	Depression	• The prevalence of depression and anxiety is particularly high in patients with autoimmune diseases, including inflammatory skin diseases such as AD.<br>• A dysregulated immune response has been linked to the precipitation of depression in many patient populations<br>• The findings, particularly the evidence from interventional clinical trials of targeted anti-cytokine therapies, suggest that pro-inflammatory cytokines associated with several skin diseases may be causally linked with the coexistent depressive symptomology
Schonmann et al [9]<br><br>Atopic Eczema in Adulthood and Risk of Depression and Anxiety: A Population-Based Cohort Study	To explore the temporal relationship between atopic eczema and new depression/anxiety	Matched cohort study	526,808 adults with AD<br><br>Matched to 2,569,030 adults without AD	Depression<br><br>Anxiety	• Adults with AD are more likely to develop new depression and anxiety<br>• AD was associated with increased incidence of new depression [HR, 1.14; 99% CI, 1.12-1.16] and anxiety [HR, 1.17; 99% CI, 1.14-1.19]<br>• For depression, a dose-response relationship with AD severity was observed

Chen et al [10]<br><br>Obsessive-compulsive disorder among patients with atopic dermatitis: a case-control study in the All of Us research program	To evaluate the association between AD and OCD in the All of Us research program	Nested, case–control study	13,433 AD cases [mean age 60, 68% female] were identified, with 53,732 matched controls.<br><br>Cases and controls were well-matched by age, sex, and race/ethnicity [all $P > 0.850$]	OCD	• Individuals with AD were more likely to have a clinical diagnosis of OCD [1.7% vs. 0.6%, $P < 0.001$]<br>• In the large, diverse cohort evaluated, there was a 2.87-fold increased odds of OCD among individuals with AD in the univariable analysis<br>• Controlling for demographic factors and comorbidities a twofold increased risk was identified
Wan et al. [15]<br><br>Neuropsychiatric disorders in adults with atopic dermatitis: A population-based cohort study	Determine risk of incident neuropsychiatric disease among adults with AD by severity	Population-based cohort study	A cohort study using electronic health records data from UK general practices from 1994 to 2015. Adults [≥18 years] with AD were matched on age, practice and index date to patients without AD.<br><br>625,083 adults with AD were compared to 2,678,888 adults without AD	Depression<br><br>Anxiety<br><br>OCD<br><br>Suicidality<br><br>ASD<br><br>ADHD<br><br>Bipolar disorder	• AD was associated with higher risk of anxiety [HR 1.14 [1.13-1.15]], depression [1.14 [1.13-1.15]] and OCD [1.48 [1.38-1.58]] across all severities.<br>• Mild or moderate AD was also associated with higher risk of autism, ADHD, bipolar disorder and suicidality.<br>• Atopic dermatitis is associated with a higher risk of multiple neuropsychiatric conditions, but these risks differ by specific condition and AD severity.<br>• Clinicians should inquire about mental health in patients with AD

Cameron et al [17]<br><br>Mind and skin: Exploring the links between inflammation, sleep disturbance and neurocognitive function in patients with atopic dermatitis.	To discuss recent findings on the putative links between AD, its association with itch, sleep disturbance and neuropsychiatric morbidity, including the role of inflammation in these conditions.	Review	NA	Sleep deprivation	• Itch was thought to predominantly drive sleep disruption in AD.<br>• Changes in sleep influence immune cell distribution and the associated inflammatory cytokines, which suggests a bidirectional relationship between AD and sleep.<br>• Inflammation is a key driver in psychological symptoms and disorders.<br>• The link between cutaneous, systemic and possible brain inflammation could at least in part be driven by the sleep deprivation and itch-driven neuronal proliferation seen in AD.
Rønnstad et al. [22]<br><br>Association of atopic dermatitis with depression, anxiety, and suicidal ideation in children and adults: A systematic review and meta-analysis.	To perform a systematic review and meta-analysis of the association between AD in children and adults and, respectively, depression, anxiety, and suicidal behavior	Systematic review and meta-analysis	NA	Depression<br><br>Anxiety<br><br>Suicidal ideation	• There was a significant association between adult AD and depression [pooled OR, 2.19; 95% CI, 1.87-2.57] and anxiety [pooled OR, 2.19; 95% CI, 1.75-2.73].<br>• AD was also associated with depression in children [pooled OR, 1.27; 95% CI, 1.12-1.45]; few data were available for anxiety.<br>• A positive association was found between AD in adults and adolescents and suicidal ideation [pooled OR, 4.32; 95% CI, 1.93-9.66].<br>• Only a few studies examined the risk of completed suicide, but the majority showed a positive association between completed suicide and AD.<br>• AD disease improvement appears to reduce mental health risks
Cheng et al. [20]<br><br>Associations of Atopic Dermatitis with Attention Deficit/Hyperactivity Disorder and Autism Spectrum	To systematically examine the existing evidence on the associations between AD, ADHD, and ASD	Systematic review and meta-analysis	Total of 24 studies with 71,373,639 subjects were enrolled	ADHD<br><br>ASD	• Significant associations of AD with ADHD [pooled OR: 1.28; 95% CI: 1.18-1.40] and ASD [pooled OR: 1.87; 95% CI: 1.30-2.68].<br>• Subgroup analyses revealed that the associations for ADHD were the most prominent in studies evaluating severe AD patients as well as in studies focusing on school-age children and adolescents [>6 years old, age, <18 years old]

Disorder: A Systematic Review and Meta-Analysis					• Among patients with AD, the pooled prevalence of ADHD was 6.6%, and the respective prevalence of ASD was 1.6%.
Zeiser et al [23]<br><br>Social and psychosocial effects on atopic eczema symptom severity - a scoping review of observational studies published from 1989 to 2019	To search for and summarize observational studies that investigated the effects of psycho-social factors on symptoms in atopic eczema and to identify research gaps	Scoping review	17 observational studies met the inclusion criteria	Depression<br><br>Stress	• Several studies found significant results for an exacerbating effect of stress on AD severity.<br>• Depression is associated with AD severity<br>• The effect of depression as a consequence of AD severity is stronger than the effect as an exacerbating factor<br>• Illness identity, anger, frustration and psychosomatic states have been found to affect AD symptoms
Holmes et al [51]<br><br>Alexithymia and Cutaneous Disease Morbidity: A Systematic Review	To clarify alexithymia's prevalence in dermatology patients and further investigate alexithymia's impact on disease burden, psychosocial comorbidities, and treatment.	Systematic review	37 publications were included	Alexithymia	• Data showed a markedly greater prevalence and severity of alexithymia in patients with AD and other skin disease compared to healthy controls<br>• Data consistently demonstrates a complex interplay between alexithymia, disease burden, and psychosocial comorbidity<br>• Identifying and addressing alexithymia in dermatology patients may improve treatment outcomes, associated comorbidities, and health-related quality of life
Kyung et al. [71]<br><br>Association of atopic dermatitis with suicide risk among 788,411 adolescents: A	To assess the association of AD and suicidal behaviors among adolescents by analyzing data from the 3rd through the 13th annual Korean Youth Risk Behavior Web-based	Cross-sectional study	A total of 788,411 adolescents completed the survey.	Suicide	• A total of 22% of adolescents had AD, with 34.7% reporting depression, 19% reporting suicide ideation, and 4.5% reporting suicide attempts.<br>• In a multivariate model, perception of stress, perceived unhappiness, and suicidal ideation were the most strongly influencing factors for depression, suicidal ideation, and suicidal attempts, respectively.

Korean cross-sectional study	Surveys [completed from 2007 to 2017]				• This study highlights the importance of evaluating mental health in patients with chronic AD
Leung et al [6]<br><br>Association of atopic dermatitis and suicide: more than a coincidence?	NA	Editorial	NA	Depression<br><br>Suicidality	• Beyond the characteristic skin inflammation of AD, there has been an increasing appreciation of its systemic effects<br>• These systemic effects may be due to the spill over of proinflammatory cytokines such as interleukin [IL]-4, IL-13, IL-17, thymic stromal lymphopoietin, IL-33, and interferon gamma from the skin into the circulation.<br>• There may be a biologic connection between systemic type 2 immune activation and mental disorders described in AD [anxiety, depression and increased risk of suicide]<br>• Therapeutic biologics used to treat AD that block the action of IL-4 and IL-13 improve quality of life and reduce depression
Vittrup et al. [88]<br><br>The association between atopic dermatitis, cognitive function and school performance in children and young adults	To examine the association between hospital-managed paediatric AD, school performance and cognitive function.	Cross-sectional study	Data was collected from the Danish national registers; between 2001 and 2019.	School performance<br><br>Cognitive function	• AD, in particular when severe, is associated with lower school performance in childhood and IQ in young men, which can interfere with academic achievements in life.<br>• Optimization of treatment of children with AD and specific educational support to children with severe AD could be needed
Pan et al [91]<br><br>Atopic dermatitis and dementia risk: A nationwide longitudinal study.	To assess the temporal association of AD with subsequent dementia.	Longitudinal study	Patients with AD aged 45 years and older [n = 1059] and 1:10 matched controls [n = 10,590] from the Taiwan National Health Insurance Research Database and	Dementia	• After adjustments for dementia-related comorbidities, patients with AD were found to be more likely to develop any dementia [HR, 2.02; 95% CI, 1.24-3.29], particularly Alzheimer's disease [HR, 3.74; 95% CI, 1.17-11.97], during the follow-up period than those in the control group.<br>• Moderate-to-severe AD was associated with a high subsequent dementia risk [HR, 4.64; 95% CI, 2.58-8.33].<br>• AD may be an independent risk factor for new-onset dementia.

			reviewed their subsequent dementia development from the enrollment date to the end of 2013		
Wen et al [92]<br><br>The link between cutaneous inflammation and cognitive impairment	To focus on the link between cognitive impairment [CI] and inflammatory skin disorders, and discuss the possible role of cutaneous inflammation in CI	Review	NA	Cognitive impairment	• The incidence of CI is higher in subjects with certain inflammatory skin disorders, including psoriasis and chronic eczematous dermatitis.<br>• Epidermal dysfunction could contribute to the development of chronic low-grade inflammation, also termed 'inflammaging', in the elderly.<br>• The evidence of CI in patients with inflammatory dermatoses suggests a link between cutaneous inflammation and CI.<br>• Because of the pathogenic role of epidermal dysfunction in ageing-associated cutaneous inflammation, improvements in epidermal function could be an alternative approach for mitigation of the ageing-associated decline in cognitive function
